# Harnessing hybrid deep learning approach for personalized retrieval in e-learning

**DOI:** 10.1371/journal.pone.0308607

**Published:** 2024-11-13

**Authors:** Sidra Tahir, Yaser Hafeez, Mamoona Humayun, Faizan Ahmad, Maqbool Khan, Momina Shaheen

**Affiliations:** 1 University Institute of Information Technology, PMAS Arid Agriculture University, Rawalpindi, Pakistan; 2 School of Arts Humanities and Social Sciences, University of Roehampton, London, United Kingdom; 3 Cardiff School of Technologies, Cardiff Metropolitan University, Cardiff, United Kingdom; 4 Department of IT&CS, Pak-Austria Fachhochschule—Institute of Applied Sciences and Technology, Pakistan; 5 Software Competence Center Hagenberg GmbH, Softwarepark, Hagenberg, Linz, Austria; University of Florida, UNITED STATES OF AMERICA

## Abstract

The current worldwide pandemic has significantly increased the need for online learning platforms, hence presenting difficulty in choosing appropriate course materials from the vast online educational resources due to user knowledge frameworks variations. This paper presents a novel course recommendation system called the Deep Learning-based Course Recommendation System (DLCRS). The DLCRS combines a hybrid Sequential GRU+adam optimizer with collaborative filtering techniques to offer accurate and learner-centric course suggestions. The proposed approach integrates modules for learner feature extraction and course feature extraction that is performed using (Embeddings from Language Models) ELMO word embedding technique in order to gain a thorough understanding of learner and course profiles and feedback. In order to evaluate the efficacy of the proposed DLCRS, several extensive experiments were carried out utilizing authentic datasets sourced from a reputable public organization. The results indicate a notable area under the receiver operating characteristic curve (AUC) score of 89.62%, which exceeds the performance of similar advanced course recommendation systems. The experimental findings support the viability of the DLCRS, as seen by a significant hit ratio of 0.88, indicating high accuracy in its suggestions.

## 1. Introduction

Traditional education methods are continuously transforming due to the rapid development of the Internet and online educational opportunities. Students and instructors are now content with the constrained traditional curriculum and would instead select courses of interest through other online education platforms [1, 2]. These platforms provide a range of benefits, including access to high-quality, specialized educational courses, comprehensive curricula with clear outlines, and supplementary tasks. Moreover, they give free courses, plentiful learning materials, and flexible learning options. Nevertheless, the rapid proliferation of platforms and courses results in a phenomenon known as "information overload, [3, 4] when learners become susceptible to confusion and decision paralysis when faced with an extensive array of options. This makes it difficult for learners to advance in their education [5, 6]. As a result, it is critical to assist consumers in swiftly selecting the correct course that interests them. Although the courses are categorized when learners utilize the online education platform to study new courses, among numerous courses, swiftly identifying engaging and personalized beneficial courses is critical.

One significance of educational recommendation systems is that they can help improve student’s learning experience and outcomes through learners’ personalized and relevant recommendations for course material [7, 8]. By tailoring course material to each student’s individual needs, interests, and learning styles, recommendation systems can help students stay engaged and motivated, leading to better retention of information and improved academic performance [9, 10]. Additionally, educational recommendation systems can help address the challenge of information overload, as they can filter and prioritize relevant course material, saving students time and effort in finding the resources they need to succeed [11–15].

Deep learning models are a potential way to solve several essential problems in context-aware course retrieval and recommendation systems. These models can make learning much better for students [16]. Deep learning models could solve many issues in context-aware course retrieval or suggestion systems. Classical recommendation systems often fall short when it comes to personalization because they don’t consider each student’s different hobbies and learning styles. Deep learning models can solve this problem because they can use a lot of data to make models that are unique for each learner [17–20]. One of the most significant issues with making sound recommendation systems is that there isn’t enough data, especially when there are a lot of possible things to offer, like educational course materials [21, 22]. Deep learning models can solve this problem by combining a more extensive range of data sources, such as demographic information, activity on social networks, and even biometric data, to make more accurate models. As students learn more, their needs and interests may change. This shows how important it is for recommendation systems to be able to adjust and update themselves in real time [23–25]. This problem can be solved using learner data on how they interact with course materials and deep learning models [26, 27]. This allows for continuous improvement and refining of suggestions [27].

Consequently, this study introduces a unique framework known as the Deep Learning-based Course Recommender System (DLCRS). This system utilizes sequential Gated Recurrent Unit (GRU) to extract learners’ actions and course properties at a comprehensive level from the provided data. The DLCRS framework can assist learners in finding their desired course contents, Addressing sophisticated and multi-dimensional sparsity challenges, and extracting more feature based information than the traditional machine learning techniques [28–30]. Adam optimizer adds value to the accuracy of retrieved results. Learner Feature Extractor (LFE) module collects learner behaviour features whereas Course Feature Extractor (CFE) module that captures course attribute feature information. LFE and CFE are integrated into the derived interaction characteristics in DLCRS. We carried out an experiment with a dataset taken from the real-world educational repository to evaluate the DLCRS framework performed and compared the results with conventional recommendation methods. The experimental findings showed that in terms of recommendation performance, the suggested DLCRS framework beats other previously practiced strategies. The following are the study’s primary contributions.

An exhaustive effort is made to analyze the state-of-the-art and existing Content Recommendation Systems (CRS) that use deep learning and machine learning prediction models in the online education context. This paper presents a specialized leaner feature extraction LFE and course feature extraction CFE module particularly which are adaptive and modular, allowing them to provide input to sequential GRU+adam based control layer for correct prediction and Collaborative Filtering (CF) based recommendation engine [31] for efficient learner-centric recommendation. The paper conducts experiments using real-world educational datasets to illustrate the efficacy of the DLCRS framework over the traditional recommendation techniques. Using a real-world dataset, we also investigated the proposed DLCRS accuracy and Hit Ratio (HR) in the online education venue.

The rest of this study is structured as follows: Section ‎2 delves into related research on Conventional Recommender Systems (CRS) and different deep-learning approaches in recommender systems. Section ‎3 delves deeply into the DLCRS framework and its layers. Section ‎4 provides the experimental evaluation and detailed outcomes of this investigation. Section ‎5 discusses the results of different experiments, while section ‎6 concludes the study, discusses its shortcomings, and suggests future research.

## 2. Related works

This section presents recommender systems based on deep learning that prioritize context-based recommendations. Context-aware recommender systems (CARS), which include contextual information in recommender systems, have arisen as a controversial subject in the industry. The deep learning approach may successfully integrate context information into recommender systems in many complicated recommendation settings.

A multimodal framework of CRS using a deep learning approach is discussed in Xinwei [32]. They proposed a course recommendation model having an Attention Mechanism and Long- and Short-Term Memory (LSTM) to overcome the issue of course recommendation. User demographic data and course rating information were combined to generate learner preferences. They conducted extensive and rigorous trials on actual datasets, and the results indicated 79% for AUC, which is promising accuracy to provide learners more accurate recommendation outcomes while course condition.

In another work, Hazrati [33] introduced a Collaborative Filtering (CF) technique based on restricted Boltzmann machines. They used deep learning in a recommendation system to find hidden qualities of persons and courses. They concluded that RBM’s CF approach has various disadvantages, including a lengthy training period and a broad range of weight values linking the hidden and visible layers, making it challenging to utilize in practical applications.

Another research [34] presented a CRS Based on DL for boosting the efficiency of user learning. Their work tried to overcome traditional recommendation challenges by introducing a novel DL-based CRS called DECOR. The proposed work gathered detailed user behaviors and course attribute data. The proposed model dealt with problems with high-dimensional data sparsity, reduced the amount of excessive information to process, and extracted information about features of high quality. By doing various tests with datasets taken from the real world, researchers evaluated the performance of their model and compared it with conventional RS. The study’s findings indicated that their model outperforms previous strategies in terms of recommendation performance.

Moreover, You discussed [35] coupled RNN with an innovative HierTCN to enable a hierarchical understanding of user inclinations and to match findings to current data in order to depict the historical effect on more recent user selections. Wan [17] proposed using learning objects and conducted their research grounded in the self-organization theory and a learner model that employed a knowledge-based approach. The system successfully addressed the cold start issue, albeit with a delay due to the presence of multiple layers of algorithms. In order to boost the RS, Trifa [36] included conceptual correlation and an adaptive key-value memorization network into a knowledge-tracing agent. Knowledge structures and recommended study material linkages were developed by Nitchot [37] with the assistance of logic, ontology, and computing.

Wu [38] used Context-aware period recommendation using RNN. Before integrating these attributes into a session-based RNN recommendation model with three layers of perceptron, the approach initially transformed contextual information into low-dimensional actual vector data. Furthermore, they integrated the functional extension module, which relies on extensive sequences. The researchers conducted comprehensive examinations on two publicly available datasets. The experimental results demonstrated that the model outperformed other state-of-the-art models regarding suggestion performance.

Sadia [39] mentioned that the critical role of the recommendation system in offering quality training resources and the deficiency of online support from services is the main reason for many difficulties. Implementing a system that provides intelligent course recommendations, considering several viewpoints, is necessary to enhance students’ competencies and knowledge. Their work offered an architecture having virtual agents to make contextual recommendations based on user preferences to help academia find relevant course materials. The experimental and statistical findings suggested that their approach increased user learning capabilities and simplified course selection based on learners’ preferences and interests.

Shanshan presented an enhanced hybrid ontology-based technique for suggesting online learning resources [46], that incorporated CF and Sequential Pattern Mining (SPM) algorithms. To avoid data sparsity and cold start difficulties, ontology was effectively leveraged for knowledge representation. The analysis of learners’ sequential access patterns in history contributed to the formulation of recommendations that align more closely with educational policies and regulations. The experimental findings demonstrated that their enhanced hybrid approach for educational material recommendations exhibited superior performance and recommendation quality compared to other pertinent techniques.

In another paper, Bhaskaran [18] employed an upgraded vector space recommender that followed the learner’s requirements, preferences and awareness level. The different ways students learn From the server blogs were put into groups and collected. After adding more preprocessing steps to make a better list of suggestions, the similarity was determined with better content-based filtering. The changed cosine similarity content was used to decide how the results were put in order. The CF method suggested putting all of the busy learners into one cluster. The proposed framework was tested using Machine Learning (ML) benchmark cases, including music, business, movie, food, book, healthcare, and Open University courses. When compared to existing, well-known methods, the simulation results of the suggested model showed better performance, precision, recall, accuracy, Mean Absolute Error (MAE), and ranking score. Some other relevant research contributions are summarized in Table 1.

**Table 1:** *Relevant research studies with dataset and evaluation parameters*.

Ref. No	Technique	Dataset	Evaluation parameters
[32]	LSTM+ Attention	Multimodal	Recall 0.79, NDCG 0.52
[8]	DNN	LMS	Precision, MAE
[7]	KNN, SVD, and NCF	Kaggle	HR 0.87
[40]	Spark	MOOC	Accuracy 0.789
[34]	ItemKNN, MF, and Neural Collaborative Filtering (NCF)	online diploma website	HR, NDCG
[18]	VSM	Open University data	MAE, Recall, Precision
[41]	CF, GSP	University data	Precision, Recall, MAE
[17]	Content-based recommendation	two courses taught by the authors	HR, Precision
[42]	Ontology-based Collaborative and content-based filtering	state universities	MAE, Precision, and Recall
[43]	Ontology-based Clustering technique and CF	COURSERA, university USMBA dataset	Precision, Recall and F1
[44]	Ontology, clustering	LMS	Learner satisfaction survey
[45]	Bi-LSTM, CNN	MOOC Cube	HR, NDCG

From the above discussion, it has been observed that several review studies based on traditional and hybrid recommendation methodologies have been published on e-learning recommender systems. These assessments explore several aspects of recommendation models, including conventional approaches, ontology-based tactics, algorithms that use machine learning, deep learning-inspired approaches, and a comparison of different recommendation strategies in the context of e-learning systems. However, our search yielded no academic instances of context-aware e-learning course recommender systems incorporating learner and course feature extraction at the input stage. This work aims to bridge the existing research gap by integrating recent studies on contextual recommenders within the e-learning domain.

## 3. Methods

**Fig 1:**
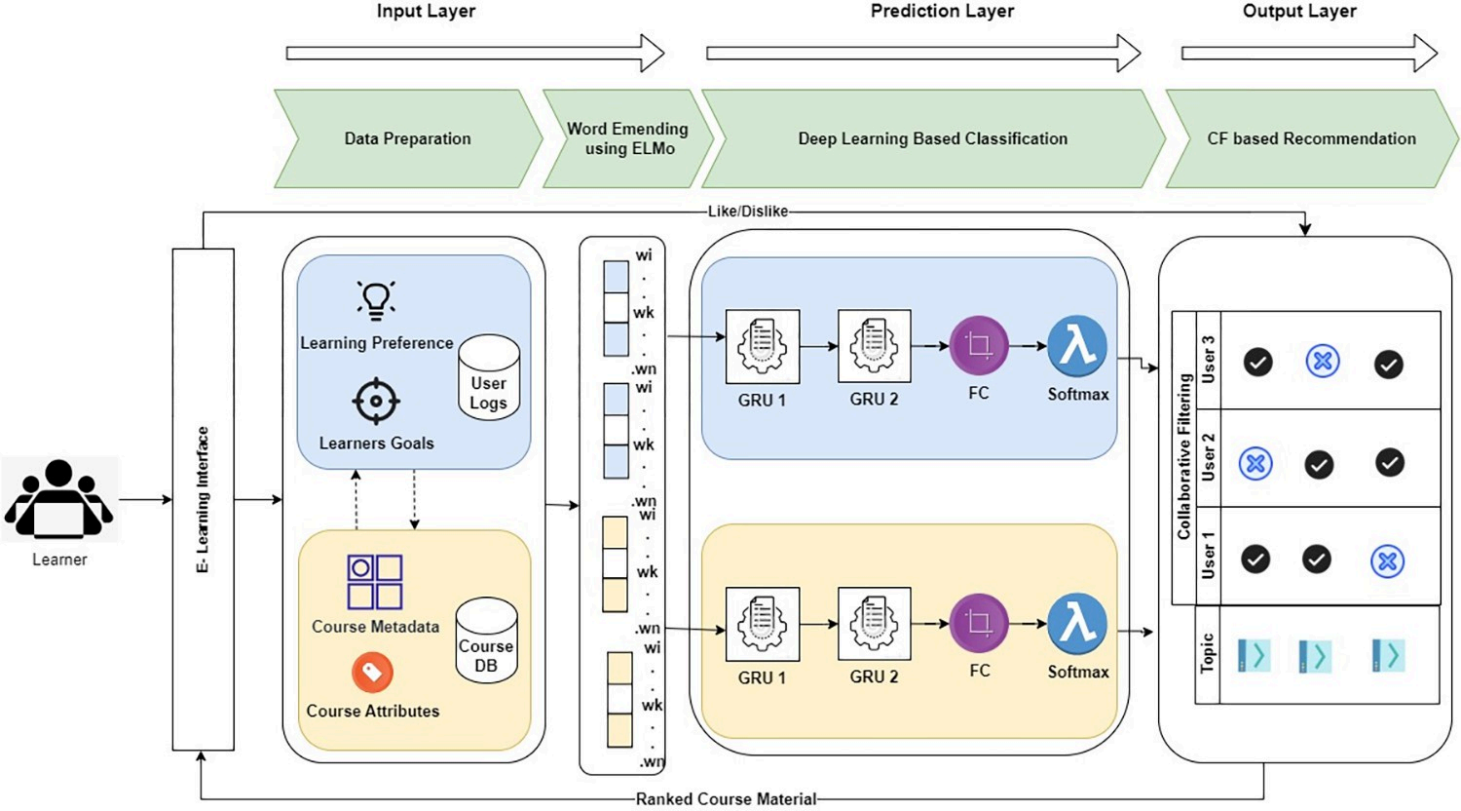
Proposed deep learning based course recommendor system.

This section describes the proposed Deep Learning-based Course Recommender System DLCRS, built on the deep learning framework and associated dataset used in this research work. This framework classifies and recommends courses based on various course and learner information. The learner’s actions while looking for and utilizing course materials are recorded and evaluated in order to provide learner-centric recommendations. The DLCRS comprises four major components: data preparation, feature embedding, deep learning course classification, and CF-based recommendation which are added in three layers. The entire process for the proposed DLCRS can be seen in Fig 1. The input layer prepares the dataset and keeps a repository of user preferences, user learning goals, and user and course logs. ELMo based word embedding is created and vector is given to control layer where sequential GRU comes in action. The final output is given to a collaborative filtering module that recommends the learning content based on the collaborative score of the end user.

### 3.1 Dataset collection

The collection and selection of data are fundamental components of any experimental study. In our research, we have utilized a benchmark dataset employed in Learning Management System (LMS) of a credible public sector university [47]. The dataset is only in the English language. The dataset included a wide range of learner profiles, course offerings, and interaction data, which offered a comprehensive and representative sample for our testing. The dataset complies with existing regulations regarding privacy and access of the LMS. The university had stringent measures and safeguards to ensure the privacy and confidentiality of learner data obtained through online learning platforms. These procedures included techniques to remove identifying information from data, mechanisms to obtain user consent, and compliance with relevant data protection rules. It was ensured that learner data is used for research purposes ethically and responsibly.

During the evaluation of the DLCRS, a significant difficulty was identified regarding the accessibility and accuracy of the datasets used to train and test the recommendation system. Although we obtained genuine dataset from a well-regarded public sector university, it is crucial to recognize the possible biases, noise, and incompleteness that are inherent in real-world data. In order to address this difficulty, we implemented thorough data preprocessing and cleaning techniques to improve the reliability and robustness of the experimental results explained in following sections. This study was approved by the University’s IRB. Informed consents were obtained from all participants online (IRB available in supporting documents). As no minors were involved in this study, parental or guardian consent was not required. In addition, we followed ethical guidelines to verify that our research complied with ethical standards and legislative regulations regarding the use of data from human subjects.

### 3.2 Input layer

**Fig 2:**
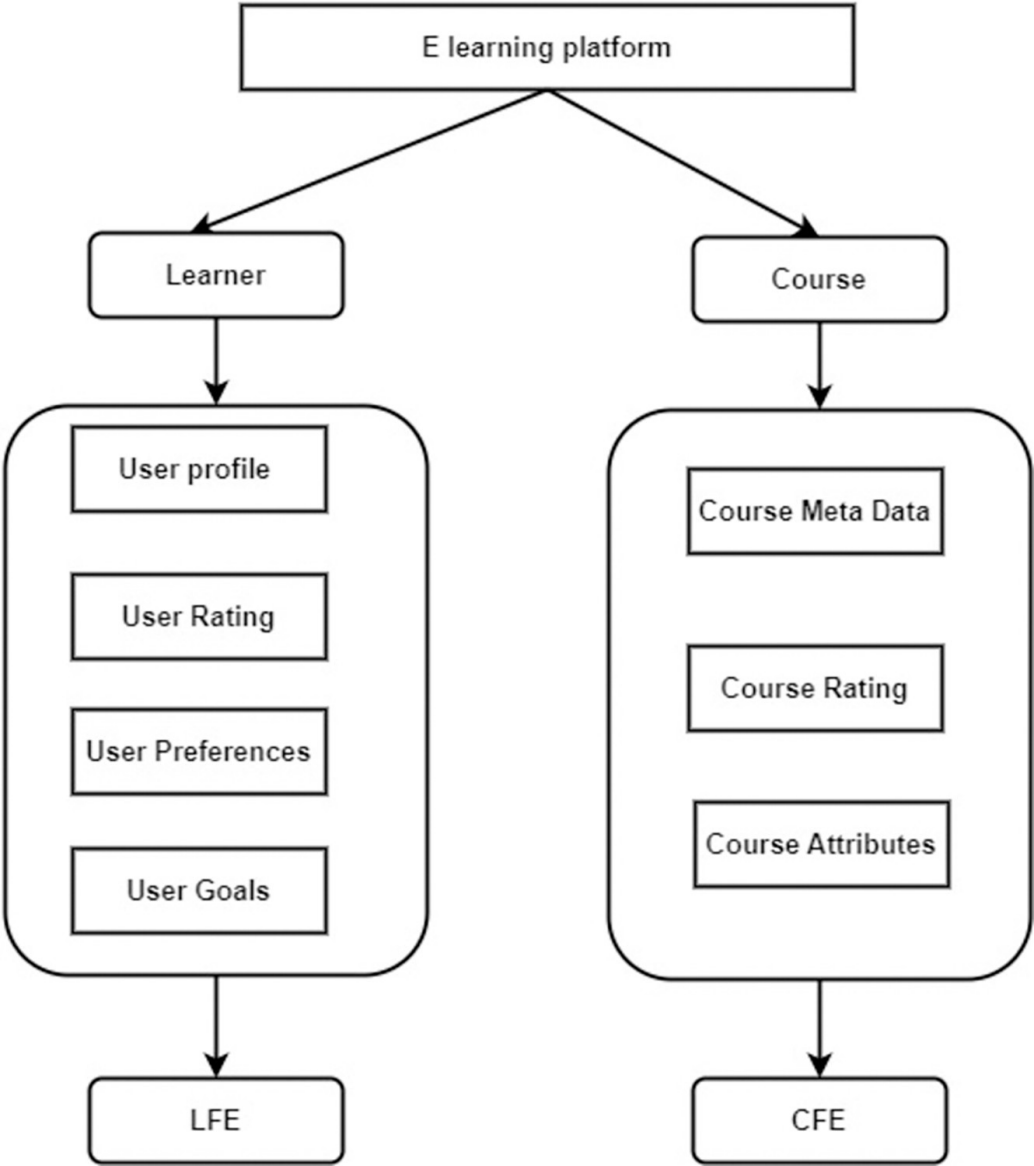
Learner and course feature extraction modules.

The e-learning site gets its information from two sources: the student feature extractor (LFE) module and the course feature extractor (CFE) module. The user profile details, learning goals, and learning choices comprise most learner data. The course metadata and course characteristics make up most of the course material. Fig 2 shows the main progression of data collection.

The ELMo (Embeddings from Language Models) approach builds word representations based on the output of a bidirectional language model [48]. The ELMo embedding for a particular token is computed as a linear combination of all layers’ hidden states in the bidirectional language model, with the linear combination’s weights learned during training:

$${ELMo}_{i}=\delta \times \sum n=0^{Lwn}\times {l_{i}}^{n}$$ (1)

Where:

*ELMo*_*i*_ = the ELMo embedding of i^th^ token in the input sequence

*L* = bidirectional language model layers count

*l*_*i*_^*n*^ = n^th^ layer hidden state of i^th^ token

*wn* = scalar weight assigned to the n^th^ layer, added at the training phase

*δ* = scalar parameter that gauges the overall scale of the ELMo vector

### 3.3 Control layer

The prediction layer employs GRU to identify short- and long-term dependencies in input text sequences [49]. Although it is possible to manage a series of text data with a single GRU layer, adding multiple GRU layers can improve model performance. The primary objective of employing a sequence of GRU layers is to enable the model to learn increasingly complex text input patterns [50, 51]. A text input is initially processed by an embedding layer, which converts each word into a high-dimensional vector. When encoded text is passed through a series of GRU layers, the material is sequentially analyzed and relationships between words and phrases are recorded. The GRU offered a simplified version of the LSTM memory cell with comparable efficacy but faster calculation speed. Each successive GRU layer acquires a distinct level of abstraction.

The initial layer may learn straightforward textual patterns, such as the occurrence of particular words and phrases. The second layer can then learn more complex patterns by combining the fundamental patterns from the first layer. This process can be replicated with multiple layers, allowing the model to discover more complicated and abstract representations of the text data. As an initial vector sequence, this study represents all characteristics of sample turnover.

$$x=\{x_{1}^{topic},x_{2}^{topic},....,x_{n}^{topic}\}$$

where *n* indicated to the number of the features in profile.

$$r_{t}=\sigma (W_{r}x_{t}+P_{r}h_{t-1}+S_{r})$$ (2)

$$u_{t}=\sigma (W_{z}x_{t}+P_{z}h_{t-1}+S_{z})$$ (3)

where σ expresses the sigmoid function. *W*_*r*_,*W*_*z*_,*P*_*r*_,*P*_*z*_ are weight parameters and *S*_*h*_ *and S*_*r*_ are bias parameters. Then, we integrated the reset gate as defined:

$${\overset{ˇ}{h}}_{t}=tanh(W_{xh}x_{t}+P_{hh}(r_{t}⨀h_{t-1})+S_{h})$$ (4)

where *W*_*xh*_,*P*_*hh*_ are weight parameters, *S*_*h*_ is the bias, and the symbol ⊙ is the element wise product, and *r*_*t*_ determines procedure to join the new input feature with the previous memory. Here *tanh* is used as activation function. Finally, gate *u*_*t*_ is updated. This determined the extent to which the new hidden state matches the old state *h*_*t*−1_ versus how much it resembled the new candidate state ${\overset{ˇ}{h}}_{t}$. The update gate *u*_*t*_ used elementwise convex combinations of ${\overset{ˇ}{h}}_{t}$ and *h*_*t*−1_. This provided the final update equation for the GRU:

$$h_{t}=u_{t}⨀h_{t-1}+(1-u_{t})⨀{\overset{ˇ}{h}}_{t},$$ (5)

Where *u*_*t*_ determines n^th^ number of previous memories are joined with the current state, and ${\overset{ˇ}{h}}_{t}$ specifies the candidate state of *h*_*t*_.

The output of the final GRU layer is fed into a fully connected (FC) layer, which learns a high-level representation of the courses. Finally, the output of the FC layer is fed into a *softmax* layer, which outputs a probability distribution over the possible course classes. Adam is renowned for its capacity for adjustable learning rates. It separately adjusts the learning rate for each parameter based on the historical gradients. This adaptability can assist in overcoming difficulties related to manually fine-tuning learning rates, which can be tiresome and ineffective. Adam is explained as follows.

Algorithm: Adam to optimize results

**Set**  Learning rate: α = 0.001

    β1 = 0.9(exponential decay rate)

    β2 =0.89 (exponential decay rate)

    ε = 1e−7

    t(current iteration step 1 to 5)

START

1 1Thefirstmomentestimate,m,foreachparameterθto0.

2 2Thesecondmomentestimate,v,foreachparameterθto0.

3 3Atimestep,t,to0.

4 4Updatetheparametersduringtraining:

  Computethegradientofthelosswithrespecttotheparameters:

  g=∇θJ(θ),whereJ(θ)isthelossfunction.

5 5Updatethetimestep:t=t+1

6 Computethefirstmomentestimate(meanofgradients):

  m=β1*m+(1−β1)*g

7 7Computethesecondmomentestimate(uncenteredvarianceofgradients):

8 8v=β2*v+(1−β2)*g^2

  Correctforbiasinthemomentestimates:

  m_hat=m/(1−β1^t)

9 v_hat=v/(1−β2^t)

9Updatetheparametersusingthemomentestimatesandlearningrate:

STOPθ=θ−α*m_hat/(sqrt(v_hat)+ε)

where, *θ* represents the neural network’s parameters (weights and biases). *α* is the learning rate, which controls the step size during parameter updates. *β1* and *β2* are the exponential decay rates for the first and second moment estimates. ε is a small constant to prevent division by zero.

*t* is the current iteration step or timestep. *g* represents the gradient of the loss concerning the parameters. *m* is the first moment estimate (the moving average of gradients). *v* is the second moment estimate (the moving average of squared gradients). *m_hat* and *v_hat* are bias-corrected moment estimates.

The parameters θ are updated using the corrected moment estimates, scaled by the learning rate and the square root of the corrected second moment estimate. The Adam optimizer adapts the learning rates for each parameter based on the historical gradient information, making it suitable for a topic classification accuracy.

### 3.4 Output layer

Once the expected course content is collected from the prediction layer, it is delivered to the CF-based recommendation layer. CF is a crucial component of the DLCRS since it leverages the combined expertise of users to provide personalized course recommendations. The DLCRS utilizes a user-based collaborative filtering method, which involves identifying users who have similar tastes and recommending courses that have received positive evaluations from these similar users. This integration encompasses multiple essential stages: 1) Computing user similarity by analyzing past interactions and preferences, 2) forming a user neighborhood consisting of users with similar preferences, 3) combining preferences within the neighborhood to determine popular courses, and 4) incorporating these combined preferences into the deep learning model, along with additional contextual information such as course descriptions and user profiles. By integrating collaborative filtering approaches, the DLCRS architecture is enhanced by supplementing the capabilities of the deep learning model. This improves accuracy and relevance in providing personalized course recommendations for individual users. The DLCRS efficiently integrates collaborative filtering with deep learning to improve recommendation accuracy by harnessing the characteristics of both approaches.

In order to obtain learner-centric course material, it is necessary to get information on the same subjects from other relevant learners. CF is beneficial in collaborative learning environments when many learners contribute comments on a single course C [9].

**Fig 3:**
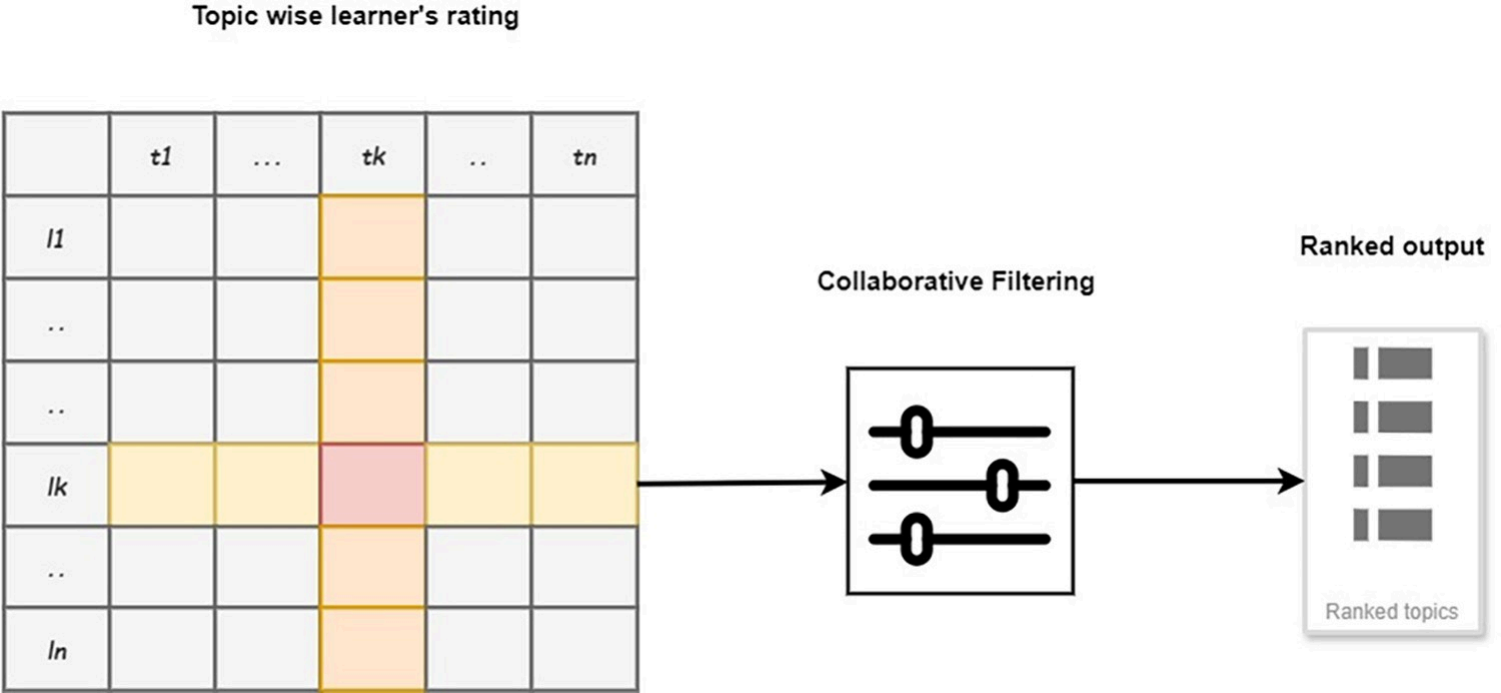
Collaborative filtering explained.


The active learner rating for any Course C might alter the ranking list of topics t for this purpose. These details are obtained from the Course rating logs that are accessible through the CFE module. To address this concern, the proposed DLCRS framework is enhanced further by incorporating CF having Rating factor ℛ, in which retrieved topic is rated by learner on a scale of 1 to 5 as shown in Fig 3. Eq (6) shows the highly rated topic *t* with user rating impact.

$$R(LP,i)=\frac{\sum l\in \begin{array}{c} LP\rho (LP,i)\times r(l,i) \end{array}}{\sum l\in LP\rho (LP,l)}$$ (6)

Where R(LP,x) indicated predicted score of topic *t* given by active learner ℓ having LP learning preference. ρ(LP,l) is Pearson correlation for LP and leaner ℓ.

The core principle of CF users is that U = u_1_, u_2_,…u_n_ rate or behave similarly concerning the subject. Topics *t = t*_*1*_, *t*_*2*_,…, *t*_*n*_ in an e-learning environment where participants rated or behaved similarly on document D’s topic t. A collection of user preferences for LOs was used by CF techniques to forecast what topics a new user might be interested in learning more about. Ratings may be expressed explicitly, as with a 1–5 scale. For instance, the list of users and their preferred or least preferred topics in Table 2 is transformed into a user-item rating matrix, as shown in Table 3, where u_i_ is the current user who gave recommendations. Participants did not indicate their preferences for particular matrix elements.

**Table 2:** User topics feedback.

User (U)	Topics
u1	(like) waterfall, Product, (dislike) service, testing
u2	(dislike) product, Web engineering (dislike)
u3	(like) Web engineering, (dislike) testing, (dislike) end user, Service (like)
u4	(like) web engineering, (dislike) Crowd Source, (dislike) Testing,
u5	(like) testing, (like) Web engineering, (dislike) testing

**Table 3:** User topics rating.

Topics (T)	Water fall	Product	Service	Testing	Web engineering	End user	Crowd source
Users	t1	t2	t3	t4	t5	t6	t7
**u1**	Like	Dislike	Dislike	-	Dislike	-	-
**u2**	-	Dislike	-	-	Dislike	-	-
**u3**	-	-	Like	Dislike	Like	Dislike	-
**u4**	-	-	-	Dislike	Like	-	Dislike
**u5**	-	Like		Dislike	Like	-	-

In this context, individuals with comparable topics and shared areas of interest are typically catered to. Every user *u* is treated as a vector in the multidimensional space of the topic *t*, and the "Pearson correlation" is used to calculate the relationships between the active user and the remainder of the users. Following the discovery of the *k* most comparable users, "the related rows in the user-item matrix R are aggregated to identify a collection of things, C, that the group has preserved together with their frequency" (in this case it is course topics). User-based CF methods then propose the top-N topic with the highest score in T that the user has not stored. As shown in Table 4. Top-N recommendations from user systems are subject to scalability and real-time performance limitations.

**Table 4:** Scores of course designers rating for different topics.

topics (Ti)	T1	T2	T3	T4	T5	T6	T7
User (ui)							
1	4	1	1	-	2	-	-
2	-	2	-	-	2	-	-
3	-	-	4	2	4	4	-
4	-	-	-	2	5	-	2
5	-	4	-	2	5	-	-

The *r*(ℓ,*x*) is score of topic *t* by leaner ℓ on the Likert scale of 1 to 5. The existing LP for topics *t* recommendation is forecasted with the help of sum of comparative rating in time *T*, shown in Eq (7). These details are obtained from the Learner rating logs that are accessible through the LFE module.


$$LP(l,i)=\frac{\sum_{T\in N}(sim(i,t))\times r_{lt}}{\sum_{T\in N}\left(||sim(i,t)||\right)}$$ (7)

The new learning preference LP is determined by taking the previous LP and the current LP and averaging them.

Pearson correlation measures the similarity *ρ*(ℓ1, ℓ2) between learners, ℓ1 and ℓ2, or *ρ*_(*t*1,*t*2)_ between two topics i and j, on the basis of correlation. The Pearson correlation coefficient assesses the linear relationship between two variables as shown in Eq (8).

$$\rho_{\left(l1,l2\right)}=\frac{\sum i\in t(r_{l1,i}-\bar{r_{l1}))}(r_{l2,i}-\bar{r_{l2})}}{{\sqrt{\sum (r_{l1,i}-{\bar{r}}_{l1})}}^{2}{\sqrt{\sum (r_{l2,i}-{\bar{r}}_{l2})}}^{2}}$$ (8)

where “i ∈ t summations are over the topics that both the learners ℓ _1_ and ℓ _2_ have rated” while “r_l_ is the average rating of the co-rated items of the i^th^ user”. Afterwards, topic relationship and similarity among learners is calculated, “the set of learners ℓ ∈ L who rated both topics t_i_ and t_j_”, the “Pearson Correlation” from Eq (9).


$$\rho_{\left(t1,t2\right)}=\frac{\sum l\in L(r_{l,ti}-\bar{r_{ti}))}(r_{l,tj}-\bar{r_{tj})}}{{\sqrt{\sum l\in L(r_{l,ti}-{\bar{r}}_{ti})}}^{2}{\sqrt{\sum l\in L(r_{l,tj}-{\bar{r}}_{tj})}}^{2}}$$ (9)

The ranked topics are then transferred to the learner dashboard as an output. By employing the cosine similarity, the degree of similarity that exists between ti and tj in terms of their rating similarity is calculated using Eq (10).


$$sim(i,j)=\frac{\sum \left(r_{l,i}-\bar{r})(r_{l,j}-\bar{r}\right)}{\sqrt{\sum {{(r}_{l,i}-{\bar{r}}_{l})}^{2}\sum {{(r}_{l,j}-{\bar{r}}_{l})}^{2}}}$$ (10)

the rating given to t sub i. by the *r*_*l*,*i*_ is rating given to *t*_*i*_ by learner ℓ *r*_*l*_ is mean rating of all rating provided by leaner ℓ.

## 4. Experimental evaluation

To demonstrate the efficacy of the proposed recommendation framework, we conducted a number of experiments and assessed our DLCRS framework using an LMS-based real-world dataset. The primary drive of the experiment was to evaluate and compare the performance, improvement in quality, and examine precision of the predictions made by the proposed DLCRS.

### 4.1 Experimental setup

Learner data is gathered for the purpose of assessment through the LMS, and this data is received through the interaction of learners with the LMS. This research makes use of a real-world dataset, which consists of 380 learners’ data, 2700 learner ratings, and 468 annotated documents of course content pertaining to the subject of software engineering. Learner data is gathered from the students attending a university that is part of the public sector. The data from undergraduate and postgraduate students who are currently enrolled in Computer Science and Software Engineering programs are contained in the learner dataset. The information for the dataset was gathered over the course of one year, notably during the COVID-19 epidemic. The code for the proposed system was implemented using the TensorFlow-Keras libraries in Python Jupiter Notebook on the Google Colab platform. The development environment utilized an Intel(R) Core(TM) i5-6200U CPU running at a frequency of 2.30GHz with 8GB of RAM. Additionally, a GPU with 12GB of memory was employed in the development process. In the conducted studies, the ratio of training data to testing data was set at 80% to 20%. The Adam optimizer was programmed using the subsequent hyper-parameters: The experiment was conducted over a total of 200 epochs, with a batch size of 64. The loss function used for this experiment was "cross entropy".

### 4.2 Evaluation parameters

The Area Under Curve (AUC) represents the area under the ROC curve, and it is the probability that a relevant course ranks higher than an irrelevant course based on its rating. Assume the suggested course list comprises C_0_ relevant course samples and C_1_ irrelevant course samples, with k relevant samples projected to be greater than k irrelevant samples; then, the AUC calculation may be described as follows:

$$AUC=\frac{k}{C_{0}.C_{1}}$$ (11)

The hit rate [35] is represented by the Hit Ratio (HR), which is the proportion of learners who have *k* suitable suggestions in the suggested course list. For each learner’s Top-N list, while the denominator T represents all test sets. The numerator *NumberHits@i* is the total of the number of test sets *i*, the HR is calculated as:

$$HR@i=\frac{NumberHits@i}{T}$$ (12)

Recall [42] relates to how much of the recommended course’s relevant course elements were accurately predicted. Recall can be written as:

$$R=\frac{Number\;of\;Relevant\;course\;content\;reterived}{Course\;content\;reterived}$$ (13)

Normalized Discounted Cumulative Gain (ℕDCG) [52] is frequently used to evaluate the recommended ranking outcomes. NDCG can be written as:

$$NDCGu@i=\frac{DCGu@i}{IDCGuu}$$ (14)

$$CNDCGu@i=\frac{NDCGu@i}{\left|u\right|}$$ (15)

*NCGu#i* indicates the discounted cumulative of the learner’s accurate list; DCGu@i is the score of the leaner’s accurate list and *C*ℕ*DCG@i* signifies the average of each learner.

## 5. Results and discussion

**Fig 4:**
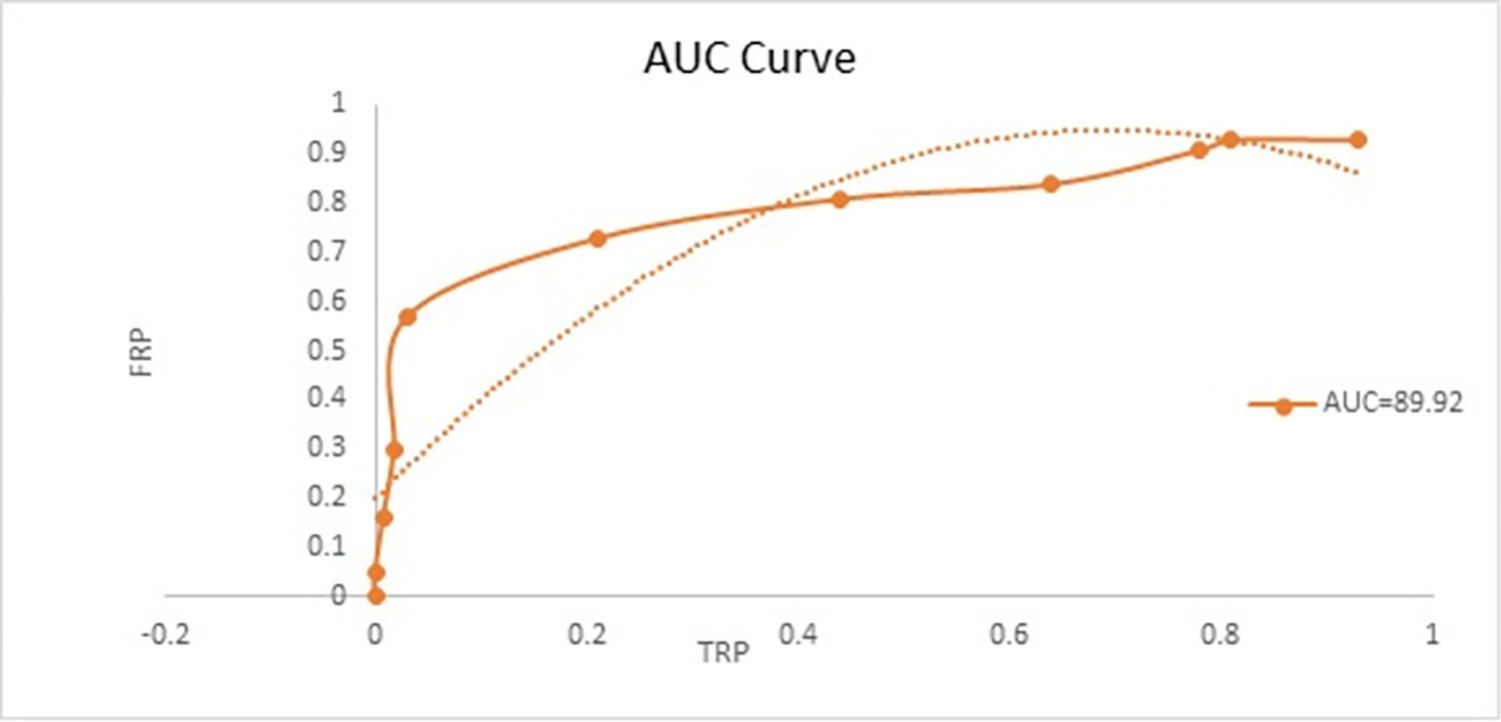
AUC of retrieved and relevant retrieved course material to demonstrate accuracy of DLCRS.

For the purpose of assessing how effective the proposed DLCRS framework was conducted in controlled environment. Learners who studied the available courses on the application accomplished this activity by browsing and clicking on the specific courses they wanted by searching for them using keywords such as the course name and suggested courses. They compiled a list of the classes they tried to access and ranked them. The platform kept a record of the various operational activities of learners and gathered explicit and implicit response data from learners in order to gain a deeper understanding of the watching patterns of learners by looking at logs. When learners accessed the material for the course, they additionally downloaded it and stored it in the appropriate directory. Through this research, we effectively improved the performance of DLCRS as a result of fusing the modal information from LFE and CFE. Additionally, in order to effectively enhance the performance of the recommendation, GRU successfully explored the important contents contained within the various contextual information as well as the progressive information that exists between courses and learners. The DLCRS framework that was presented in this paper achieved better course recommendation results, with the AUC score reaching 89.62% and the hit rate reaching 88% topic wise and 94% learner wise; indicating that the proposed DLCRS framework is able to improve desired course recommendation accuracy effectively. The experimental results show that the proposed DLCRS framework was able to achieve enhanced results as shown in Fig 4. An AUC score of 89.62% indicates that the DLCRS framework has a high ability to distinguish between positive and negative outcomes. In other words, it can accurately suggest courses that match learners’ interests and preferences. A higher AUC value signifies that the DLCRS has a superior capability to accurately suggest appropriate courses to users, taking into account their preferences and interactions. This suggests that the DLCRS is proficient in precisely capturing and utilizing the implicit and explicit reactions of learners, as well as their browsing and interaction practices, to provide customized course recommendations.

**Fig 5:**
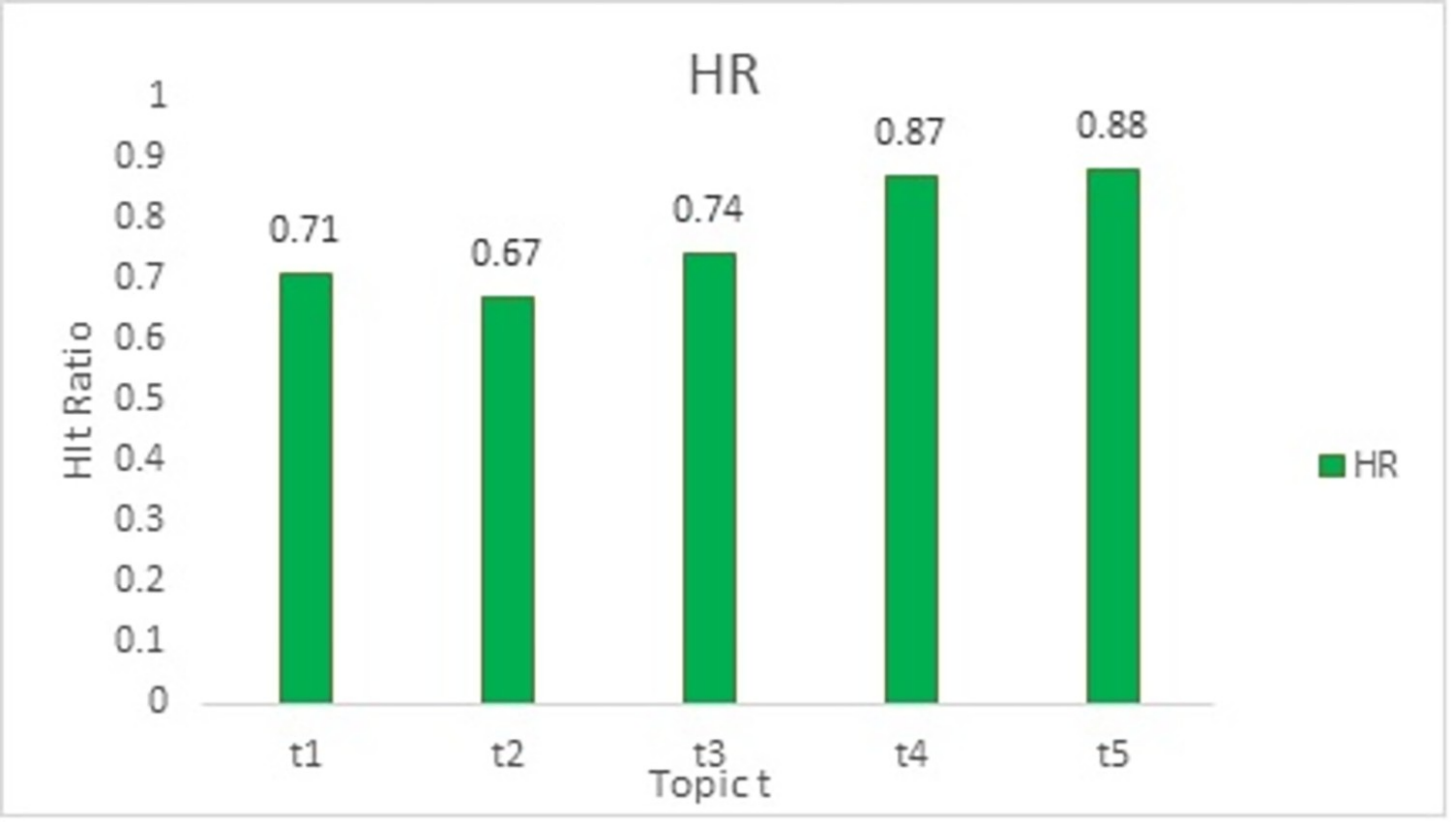
Topic wise hit ratio.

In order to assess the usefulness of the suggested course materials, we conducted an analysis and experimentation to test the impact of various topic feature combinations on the outcomes. Fig 5 depicts a comparison of information hit rates across five topics for a specific course.

**Fig 6:**
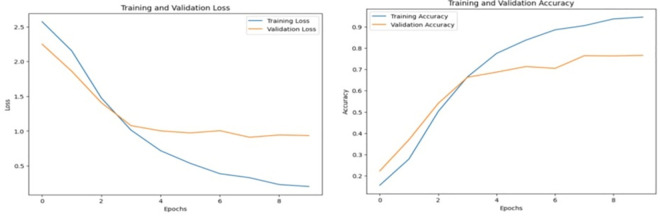
Accuracy and validation over 10 epoch.

The experimental results indicate that t2 has the lowest hit rate, suggesting that it contains the least amount of relevant information. T5 has the maximum HR for the topic, which is 88 percent. When the hit rate of t3 is compared to the HR of t4, it is found that course-related information enhances the accuracy of the recommendation, indicating that t4 contains more course-related information. Thus, it is demonstrated that contextual course topic information improves the accuracy of recommendation. Fig 6 reflects the validation loss and validation accuracy over 10 epochs during training. Training and validation accuracy graphs are essential machine learning tools for model performance and generalization evaluation. These graphs show a model’s accuracy metrics on the training and validation datasets across epochs.

Training accuracy in Fig 6 shows how well the model learns training data patterns. As the model iteratively adjusts its parameters to minimize training dataset mistakes, accuracy increases. Training accuracy may rise substantially if the model learns the dataset well. The validation accuracy graph shows how effectively the model generalizes to unseen data (validation set) during training. It helps determine model overfitting or underfitting. If validation accuracy falls behind training accuracy or stagnates while training accuracy rises, the model is overfitting and memorizing the training data rather than learning its patterns.

**Fig 7:**
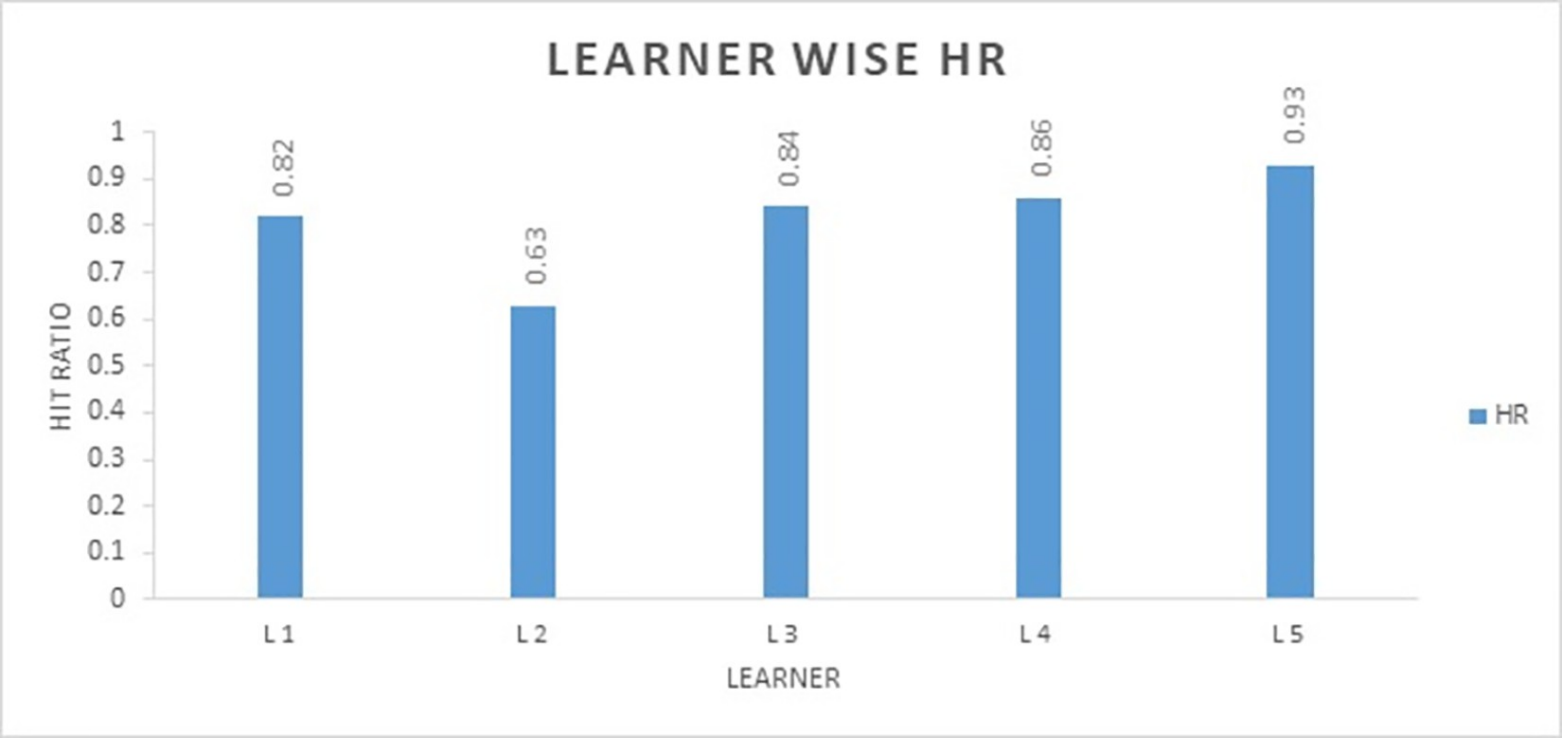
Learner wise hit ratio.

The demographic characteristics of learners yield a limited amount of information and have a low success rate in relation to course outcomes. Including learners’ explicit feedback and scoring record in the form of content rating as well as implicit feedback data (browsing activities on the course) may significantly increase user hit rate. The above experimental findings suggest that combining different user information boosts course hit rate and improves course recommendation effect. Fig 7 shows the hit rate of different learner features.

**Fig 8:**
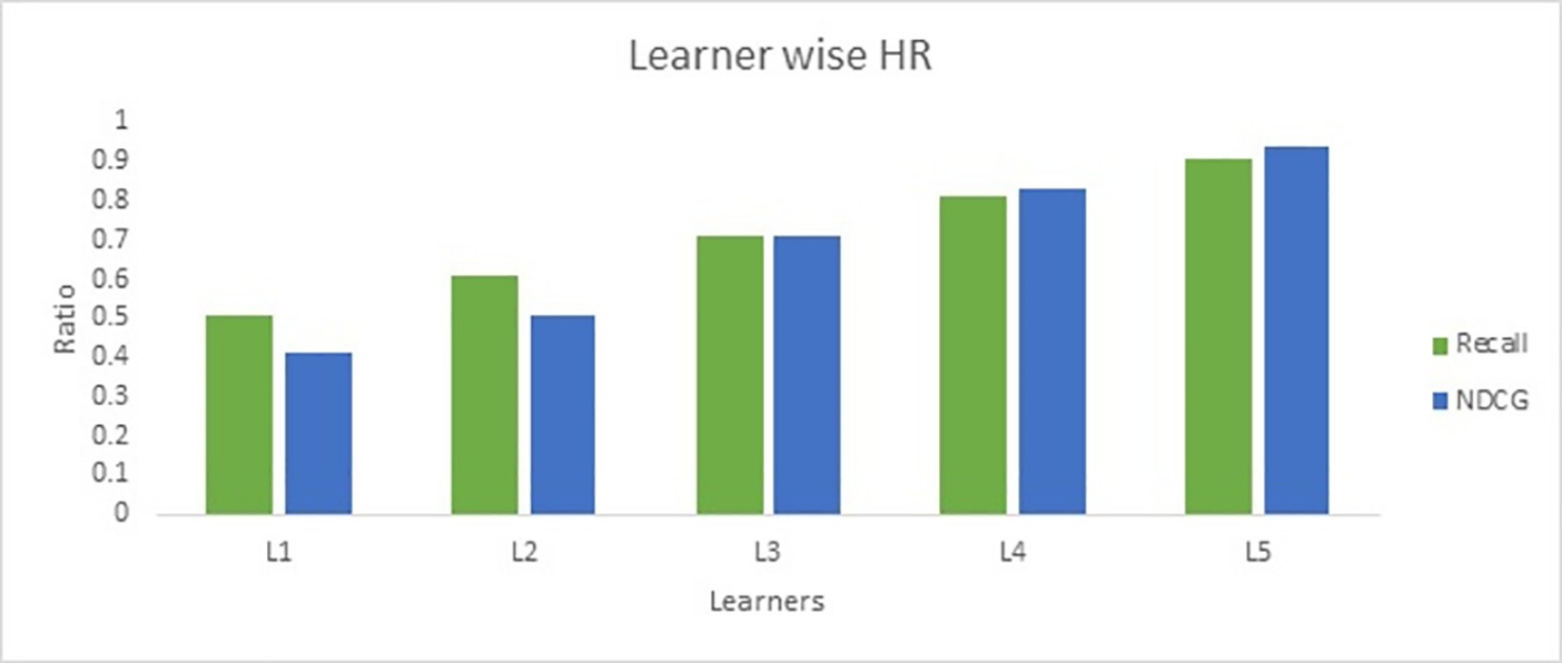
Recall and NDCG of proposed DLCRS.

Similarly, the ranking of recommended course contents improved with HR increase. As shown in Fig 8, the HR reached 90% as contextual information increases the information gain. At the same time, the NDCG index of ranked course information increase with HR boost. The increase in recall and NDCG indicate that recommendation of contextual information for the specified topic for learners is improving.

The comparison of performance metrics is carried out using AUC, NDCG, HR, Recall and Precision. The performance evaluation uses metrics namely Precision, F-measure and Recall to assess the precision and efficiency of the proposed DLCRS. Table 5 explains the collective assessment of proposed DLCRS with state of art recommendation systems.

**Table 5:** Evaluation of DLCRS with existing CRS.

Dataset	Source	Method	Recall	HR	NDCG	AUC
DS 1 [32]	Educational Dataset	LSTM+AM	0.58	0.48	27%	0.53
DS2 [34]	LMS	ItemKNN, NCF	0.56	0.52	53%	0.58
DS3 [45]	MOOC Cube	Bi-LSTM, CNN	0.43	0.81	65%	0.71
**DLCRS**	**LMS**	**Sequential GRU +Adam, CF**	**0.91**	**0.88**	**94%**	**0.89**

**Fig 9:**
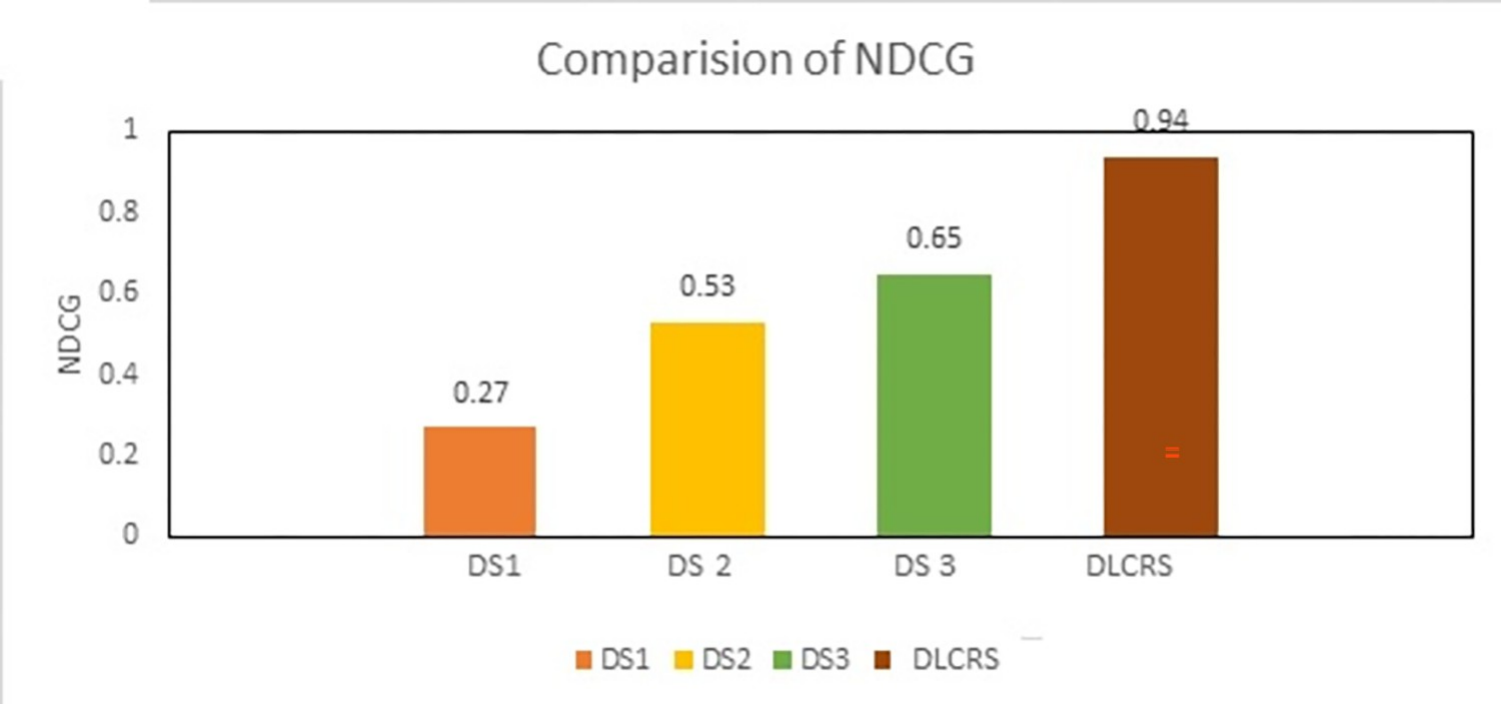
Comparison of NDCG among existing approaches and proposed approach.

The dataset DS1 [32] has used LSTM and AM based framework and content-based datasets acquired from educational sites were utilized. The AUC is 0.53, the recall is 0.58, HR is 0.79%. Similarly, the dataset DS2 [34] after experimenting with a publicly available data set from Korean LMS. In this research, collaborative neural filtering is employed. 0.56 recall, 0.58 AUC, 0.52 HR is achieved. Moreover, the dataset used in [45] is abbreviated as DS3, acquired dataset from publicly available MOOC utilizing bi- LSTM and CNN approach. Calculated AUC was 0.71, recall was 0.43, NDCG was 0.59, and HR was 0.81. Fig 9 demonstrates the comparison among existing approaches and proposed one.

**Fig 10:**
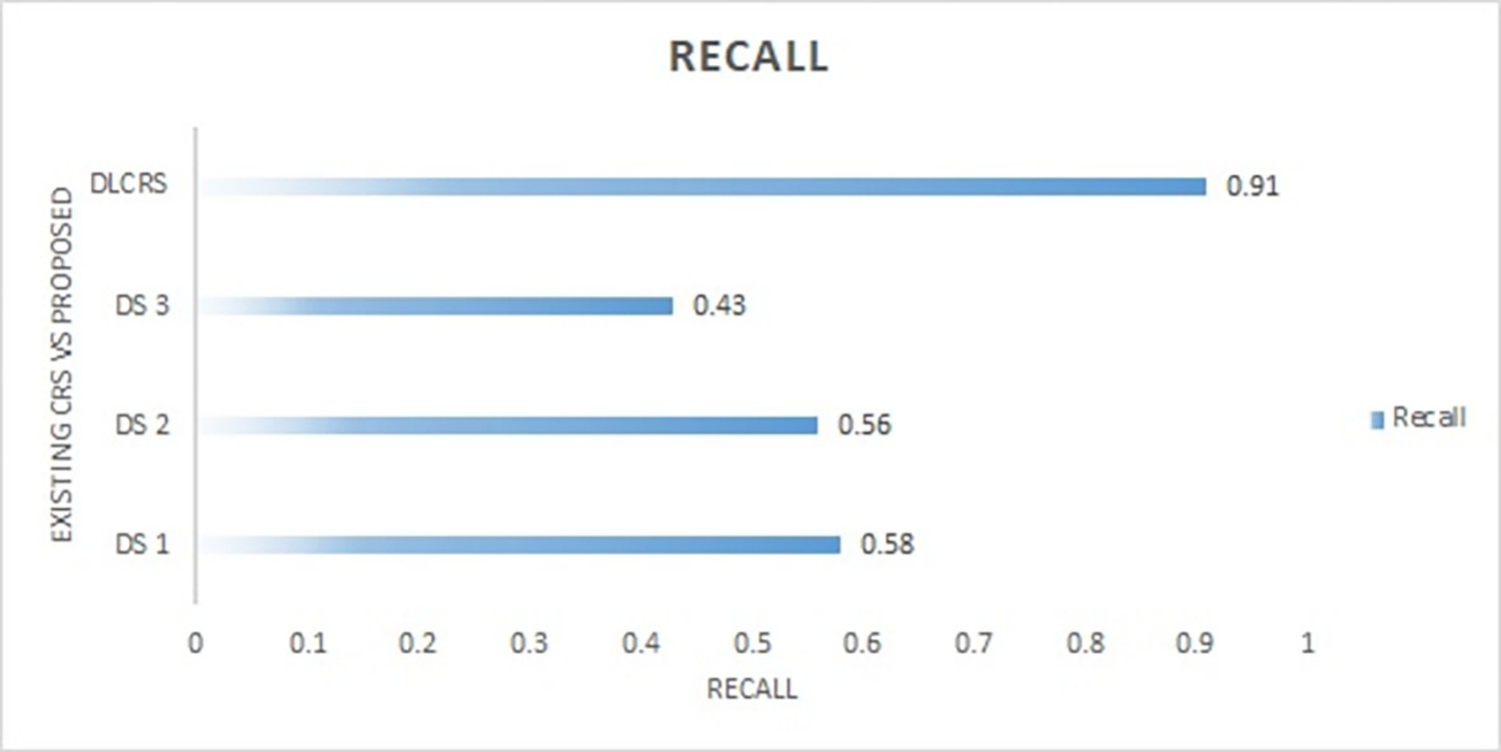
Collective Recall and NDCG comparison of DLCRS with other CRS.

As shown in Fig 10, DS1 has lower AUC and recall as compared to DLCRS. The proposed recommender system is efficient as its accuracy is higher than DS1. Similarly, DS2 has lower HR and accuracy from proposed dataset. The recall of proposed system is higher than DS2. When compared with DS3, the proposed system outperforms DS3 with higher AUC and recall. Collectively, proposed system is found to be more efficient and productive because of higher HR.

The results of experiments have revealed necessary online learning platform improvements: First, the DLCRS emphasizes personalized course recommendations for individual learners. Online systems must be flexible to meet varied learner preferences and styles. Deep learning and collaborative filtering enable personalized, engaging, and satisfying learning. Second, the DLCRS’s correct course recommendations enhance users’ learning. This shows how advanced recommendation algorithms simplify course material access. Platform recommendation algorithms that consider learner preferences and interactions can boost user happiness and retention. Our DLCRS experiments reveal learner behavior and preferences, enabling data-driven online platform decision-making. Administrators can refine course offerings, identify areas for improvement, and adjust content to user needs by analyzing feedback and performance analytics. Finally, our repeated experimentation emphasizes online platform design’s need for continual improvement. Develop recommendation algorithms, content strategies, and user interfaces using feedback mechanisms and performance indicators to improve effectiveness and user experience. To conclude the discussion, the proposed DLCRS framework has a lot of potential in terms of the recommendation of courses that are user-friendly and efficient due to the speed with which it operates. Learners have the ability to access and make use of these resources by choosing a variety of course resources, lesson plans. Moreover, the retrieved course materials are contextually related to one another. In conclusion, the intuitive user interface of the proposed DLCRS is adaptive at catering the needs of the learner that even a novice would feel at ease using it while accessing course content.

### 5.1 Implication of the study

The DLCRS paradigm has substantial implications in the realm of recommendation systems, especially in e-learning contexts. Enhanced personalization has facilitated the retrieval of course resources that closely match user preferences, leading to practical applications. By identifying more appropriate course materials, learners can achieve greater educational achievements. Analyzing information acquired from learners’ preferences can assist e-learning platforms in determining course offerings, designing content, and enhancing e-learning platform services. The incorporation of contextual recommendations enhances the effectiveness of collaborative filtering and personalization in e-learning by utilizing learners’ interactions, thereby introducing a fresh perspective on improving the accuracy of e-learning suggestions. Lastly, DLCRS enhances the existing expertise on the application of deep learning techniques in educational recommendation systems.

### 5.2 Limitations

While the DLCRS technique has shown encouraging outcomes, it is important to acknowledge a few limitations. As learners’ interests and requirements may change over time, this evolution can impact their learning preferences. Currently, the proposed DLCRS does not consider the temporal changes in the learner’s preferences. In addition, both new learners and new course material may encounter the cold start problem, which might impact their recommendation and ranking. To enhance the effectiveness and capability of DLCRS, it is important to solve the critical issue of cold start and temporal changes in future.

## 6. Conclusion

This study aimed to propose a hybrid deep neural network that integrates GRU sequences and heterogeneous features of learners’ preferences. The objective was to enhance e-learning platforms by enabling collaborative filtering for recommending course content. This is important because both the sheer number of students and the number of online classes are growing at a swift rate. First, we focused on using a word embedding approach based on the ELMo for obtaining a high-dimension vector of features and initialized learners as a comparable group. The top-N probable courses are then generated using sequential GRU, synchronous sequences having heterogeneous profile characteristics. Course topics are recommended to learners via collaborative filtering. Our study aimed to offer context-aware course content to participants based on their learning preferences.

The main contribution of this study was the incorporation of sequential GRU with the integration of diverse features leading to enhanced collaborative filtering. In addition, ELMo word embedding was utilized to enhance feature representation. Furthermore, the DLCRS approach suggests customized LOs, resulting in improved learning resources for learners. The proposed DLCRS surpasses existing personalized e-learning recommendation systems with NDCG and AUC scores of 94% and 89% respectively.

However, several restrictions must be overcome in order for it to progress:

- To solve learners’ cold start issue using deep learning and sentiment analysis
- Combine the method with more advanced and precise clustering algorithms such as bi-LSTM.

Furthermore, future research should look at creating sentiment-based feedback on course development models that use user input and learners’ history sequence data, as well as developing a recommendation model for higher education courses in the multi-context domain. In future studies, we also plan to explore the scalability of the DLCRS by evaluating its performance effectiveness with increasingly large and diverse datasets.

## Supporting information

S1 Data (XLSX)
